# Capecitabine-Induced Severe Refractory Diarrhoea Managed With Methylprednisolone: A Case Report

**DOI:** 10.7759/cureus.77821

**Published:** 2025-01-22

**Authors:** Tanmay Jain, Itish Patnaik, Sweety Gupta, Rohit Gupta, Vanisha Pundir

**Affiliations:** 1 Gastroenterology and Hepatology, All India Institute of Medical Sciences, Rishikesh, Rishikesh, IND; 2 Radiation Oncology, All India Institute of Medical Sciences, Rishikesh, Rishikesh, IND

**Keywords:** acute severe colitis, capecitabine-induced colitis, capecitabine induced diarrhea, capecitabine-induced diarrhoea, chemotherapy-induced complications, chemotherapy-induced diarrhoea, chemotherapy-related toxicity, colitis-associated cancer

## Abstract

Chemotherapy-induced diarrhoea (CID) is a common adverse effect, especially with capecitabine. The majority of cases are mild. Current guidelines have recommended loperamide and octreotide for severe CID. Steroids have not been included in the current guidelines. We present a case of treatment-resistant capecitabine-induced diarrhoea, successfully managed with methylprednisolone.

## Introduction

Chemotherapy-induced diarrhoea (CID) is a prevalent and challenging complication in patients undergoing cancer treatment, particularly with agents like capecitabine, an oral prodrug of 5-fluorouracil (5-FU) [1]. Chemotherapy-induced diarrhoea can vary in severity and has the potential to significantly disrupt treatment plans due to its impact on patient well-being, leading to dose reductions or discontinuation of chemotherapy.

Capecitabine is commonly used in the treatment of colorectal and breast cancers [2]. Thymidine monophosphate is the active form of thymidine, which is required for the de novo synthesis of DNA. 5-fluorouracil is a thymidylate synthase inhibitor. Capecitabine gets converted into 5-FU. This conversion occurs more intensively in tumour cells due to higher levels of the enzyme thymidine phosphorylase, which targets cancer cells selectively. Once converted, 5-FU exerts its effect by inhibiting the enzyme thymidylate synthase. By blocking thymidylate synthase, 5-FU prevents DNA synthesis, leading to impaired cell division and, ultimately, cell death [3]. Additionally, 5-FU is incorporated into RNA, disrupting RNA processing and function, which contributes further to its cytotoxic effects [3].

However, one of the limiting side effects of capecitabine therapy is severe CID, which can necessitate medical intervention. The management of CID typically involves supportive care, including fluid and electrolyte replacement, and pharmacological interventions such as antidiarrhoeal agents (e.g., loperamide) and, in more severe cases, octreotide [4]. However, not all patients respond to these standard treatments, and alternative therapies must be considered.

Corticosteroids, which have potent anti-inflammatory properties, have been used in cases where conventional treatments have failed [5]. While corticosteroids are not routinely used for CID, they can be effective in cases with a significant inflammatory component, as evidenced by the patient’s response in this case. The use of methylprednisolone in this context is supported by its ability to modulate the immune response and decrease intestinal inflammation, thereby reducing the frequency and severity of diarrhoea.

While severe refractory diarrhoea caused by capecitabine is uncommon, recognising the early signs of treatment resistance and the potential underlying inflammatory component is crucial for timely intervention. This case highlights the importance of integrating clinical findings, such as persistent diarrhoea unresponsive to standard therapy and endoscopic evidence of inflammation, into the diagnostic process. By doing so, clinicians can identify patients who may benefit from alternative approaches, such as corticosteroid therapy, even when it falls outside the current guidelines.

This case report explores the therapeutic challenges associated with managing severe CID in a patient with rectal adenocarcinoma treated with capecitabine and highlights the successful use of methylprednisolone.

## Case presentation

A 34-year-old male patient with no significant past medical history was diagnosed with adenocarcinoma of the rectum, staged as T3 N2a M0. The patient presented with loose stools occurring 10-15 times per day, in small quantities, without associated pain, four days after initiating chemotherapy with capecitabine (2,500 mg/day). There was no reported history of haematochezia or evidence of malabsorption. There was no concomitant treatment. Examination revealed a pulse rate (PR) of 81 beats per minute and blood pressure (BP) of 103/72 mmHg. Abdominal and systemic examinations were within normal limits. The patient was admitted for grade 3 diarrhoea according to the Common Toxicity Criteria [6].

**Figure 1:**
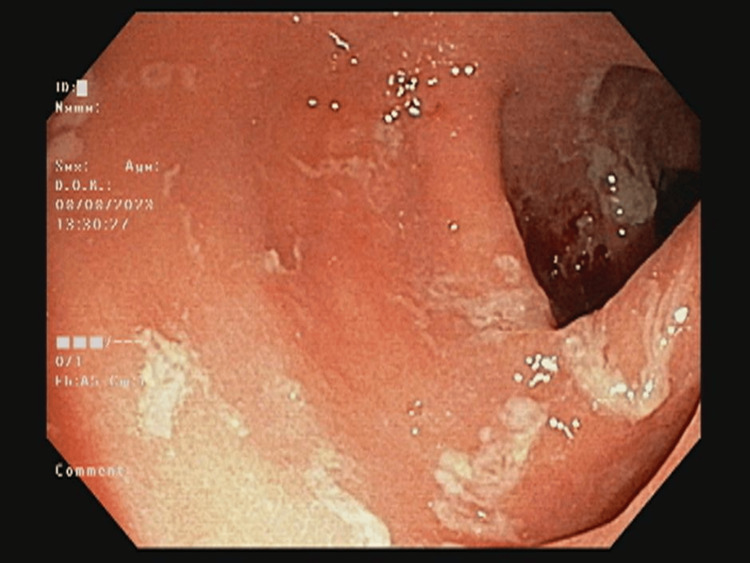
Colonoscopy showing descending colon with oedematous mucosa, indicative of significant inflammatory involvement

**Figure 2:**
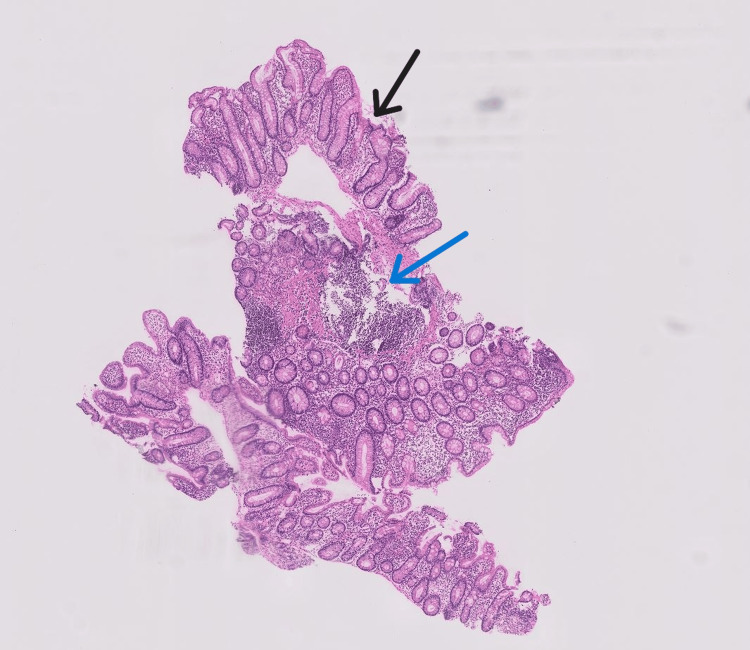
Histopathology (low magnification) from the colon showing crypt distortion (black arrow) and inflammatory infiltrates (blue arrow). These changes suggest severe inflammation, correlating with the patient's refractory diarrhoea and justifying corticosteroid treatment.

**Figure 3:**
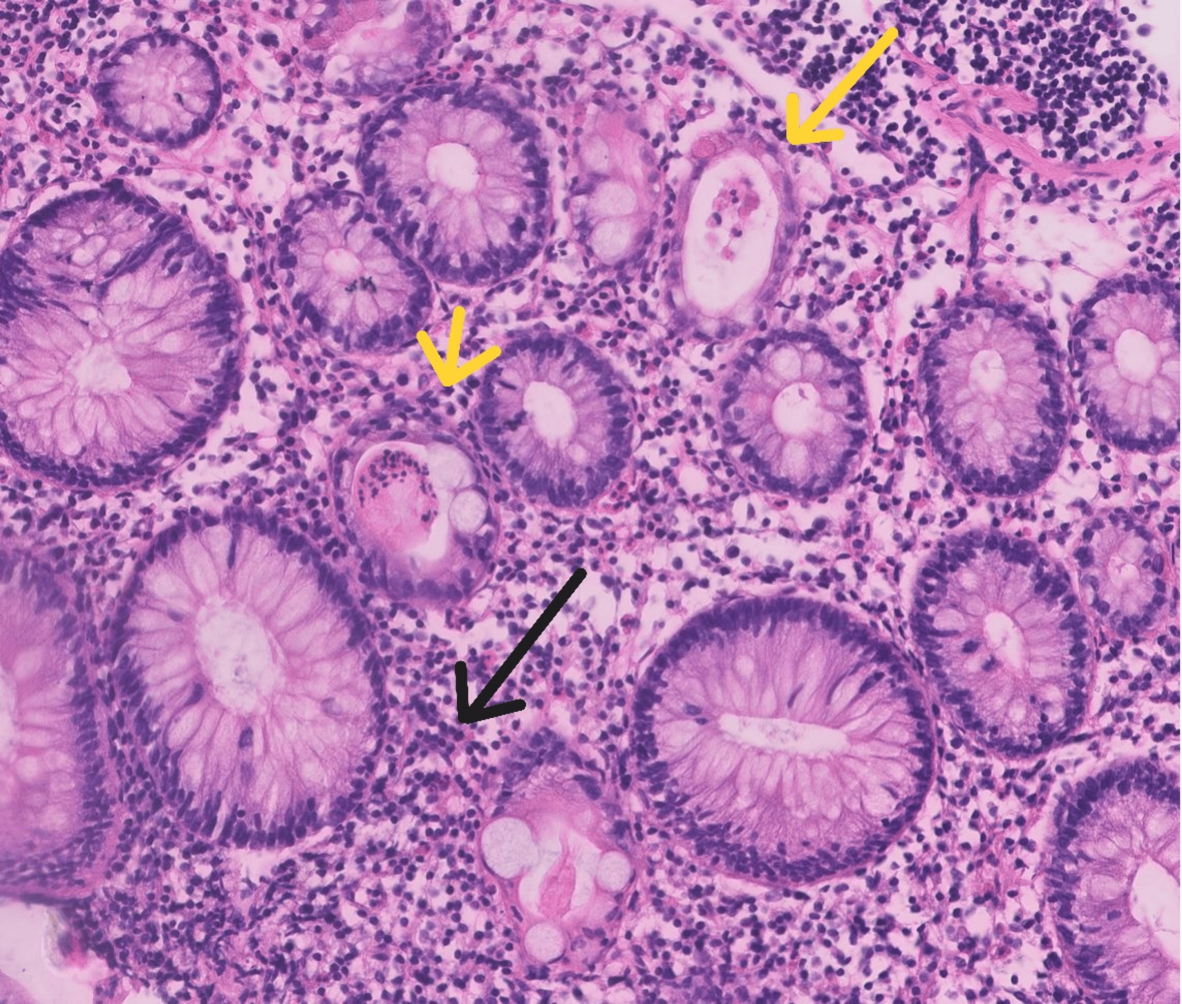
Histopathology image (magnified) showing crypt abscess (yellow arrow) and inflammatory infiltrate (black arrow). These findings confirm an inflammatory component in the diarrhoea, guiding the use of methylprednisolone.

Baseline investigations are mentioned in Table 1. Stool microscopy and culture were negative. The stool test for *Clostridium difficile* glutamate dehydrogenase (GDH) and enzyme-linked immunosorbent assay (ELISA) was negative. Dihydropyrimidine dehydrogenase deficiency could not be tested due to financial issues. A colonoscopy revealed ulcero-proliferative growth in the rectum. Oedema and erythema were noted from the rectum to the ascending colon (Figure 1). The caecum and terminal ileum were normal. Four bits were taken from each segment of the colon. Histopathology revealed crypt distortion and a mixed inflammatory infiltrate with increased basal plasma cells (Figures 2-3). A contrast-enhanced computed tomography (CECT) of the abdomen showed mesorectal fat stranding in the rectum and sigmoid colon with a growth in the rectum.

**Table 1:** Laboratory investigations on admission ALT: alanine aminotransferase; AST: aspartate aminotransferase; ALP: alkaline phosphatase; TSH: thyroid-stimulating hormone, TLC: total leucocyte count

Parameters	Values	Reference values
Haemoglobin	14.9 g/dL	13.0-17.5 g/dl
Haematocrit	42.9 %	38.3 -48.6%
TLC	5.18 10^3/uL	4,000-11,000^3/uL
Platelet count	366 10^3/uL	150- 450^3/uL
Sodium	130 mmo/L	135-140 mmo/L
Potassium	3.8 mmo/L	3.5- 5.2 mmol/L
Urea	46.0 mg/dL	8–24 mg/dL
Creatinine	0.87 mg/dL	0.7–1.2 mg/dL
Total bilirubin	0.73 mg/dL	0.3- 1.2 mg/dL
Direct bilirubin	0.19 mg/dL	<0.3 mg/dL
AST	23.0 U/L	8-35 U/L
ALT	13.0 U/L	7-35 U/L
ALP	64.0 U/L	44-120 U/L
Total protein	5.0 g/dL	6.0- 8.3 g/dL
Albumin	3.0 g/dL	3.4- 5.4 g/dL
TSH	3.527 µU/mL	0.4- 4.5 µU/mL

Supportive treatment with IV fluids, probiotics, and racecadotril was initiated. Initial treatment with ciprofloxacin and metronidazole was ineffective. A brief trial of nitazoxanide was also started. Following a 48-hour trial of loperamide, octreotide 100 mcg subcutaneously three times daily (TDS) was initiated for four days and increased to 200 mcg TDS for three days due to lack of improvement. Partial relief was achieved, with a decrease in stool frequency to six to eight times per day. Due to the slow response, methylprednisolone 8 mg TDS was introduced, resulting in a marked improvement in symptoms within two days. Budesonide was not given due to financial issues.

The patient was discharged on a tapering dose of steroids over 10 days and followed up in the outpatient department. At the last follow-up, he reported a complete resolution of symptoms and has remained asymptomatic since.

## Discussion

The management of CID is a critical aspect of cancer care, particularly for patients treated with capecitabine, which is known to cause gastrointestinal side effects. In this case, the patient’s CID was severe and unresponsive to standard treatments, including antibiotics, octreotide, and loperamide, which are typically first-line therapies for CID. The use of methylprednisolone in this context is noteworthy, as corticosteroids are not standard treatment for CID due to their potential for systemic side effects and immunosuppression.

One randomised controlled trial (RCT) elucidated the role of budesonide in 5-FU-induced severe diarrhoea (1). However, no RCTs have been conducted for capecitabine-induced diarrhoea. There have been two case reports of the use of budesonide in terminal ileitis and colitis caused by capecitabine [5-6].

The decision to use methylprednisolone was based on the patient’s refractory symptoms and the presence of significant inflammatory changes in the colon, as evidenced by colonoscopy and histopathology findings. The rapid improvement in symptoms following the initiation of methylprednisolone suggests a substantial inflammatory component to the patient’s CID, which was amenable to steroid therapy. This aligns with the known anti-inflammatory properties of corticosteroids, which can modulate the immune response and decrease intestinal inflammation.

The use of antibiotics in this case was initially aimed at addressing potential infectious causes of diarrhoea, a critical step in the diagnostic workup of CID. Stool cultures and *Clostridium difficile* testing ruled out infections, further supporting an inflammatory aetiology. This sequential approach of excluding infectious causes and identifying inflammatory components highlights the importance of tailoring treatments to the underlying pathophysiology of CID rather than relying solely on symptom management.

The successful management of this patient’s CID with methylprednisolone may have broader implications for the treatment of severe capecitabine-induced diarrhoea. While the use of corticosteroids in CID requires careful consideration due to the risk of adverse effects, this case demonstrates that they can be a valuable option in specific clinical scenarios where conventional therapies fail. It also highlights the importance of individualised patient care and the need for clinicians to be adaptable in their treatment approaches.

This case underscores the need for vigilance when managing patients on capecitabine therapy who present with severe diarrhoea, particularly if unresponsive to loperamide, antibiotics, or octreotide. Clinicians should consider performing endoscopic evaluation in cases of refractory symptoms to assess for inflammatory changes, as this can guide the initiation of targeted therapy such as corticosteroids. Key diagnostic clues include persistent symptoms despite standard management, histopathological evidence of inflammation (e.g., crypt distortion and basal plasma cell infiltration), and the exclusion of infectious causes like *Clostridium difficile*. Identifying these features can help clinicians tailor treatments to the underlying pathophysiology rather than solely addressing symptoms. While corticosteroids are not first-line treatments for chemotherapy-induced diarrhoea, their potent anti-inflammatory properties may provide significant benefit in select cases, as seen here.

Furthermore, this case highlights the importance of contextualising treatment choices within the broader framework of CID management. The sequential approach, ruling out infectious causes, assessing for inflammatory findings, and considering corticosteroids only when standard therapies fail, provides a practical guideline for clinicians managing similar cases. By documenting the rationale behind each treatment step, this case offers insights into the decision-making process that may be applicable in other challenging scenarios.

This case contributes to the limited but growing body of literature on the management of CID with corticosteroids and underscores the need for further research. Prospective studies are needed to better understand the role of corticosteroids in CID and to establish guidelines for their use in this context.

## Conclusions

In conclusion, this case highlights the challenges associated with managing severe refractory capecitabine-induced diarrhoea, a rare but debilitating complication of chemotherapy. Despite the use of standard therapies, including antibiotics, loperamide, and octreotide, the patient’s symptoms persisted, necessitating an alternative approach. The successful use of methylprednisolone underscores the potential role of corticosteroids in managing cases with significant inflammatory components, as evidenced by endoscopic and histopathological findings. This case emphasises the importance of individualised treatment strategies in oncology, particularly for adverse effects that do not respond to conventional therapies. Further research, including prospective studies, is warranted to better define the role of corticosteroids in the management of chemotherapy-induced diarrhoea and to establish evidence-based guidelines for such cases.
